# Supplementary material for: DMRforPairs: identifying Differentially Methylated Regions between unique samples using array based methylation profiles
Source: BMC Bioinformatics. 2014 May 15;15:141. doi: 10.1186/1471-2105-15-141 (PMC4046028; doi:10.1186/1471-2105-15-141)
Supplement: Additional file 2 — DMRforPairs output for the comparison of A431-MCF7 and NA17018-NA17105. Please start from the HTML files in each folder. Available via the BMC Bioinformatics website. [file 1471-2105-15-141-S2.zip › 1394847754114233_MOESM2_ESM/A431_MCF7/figures/28260.pdf]

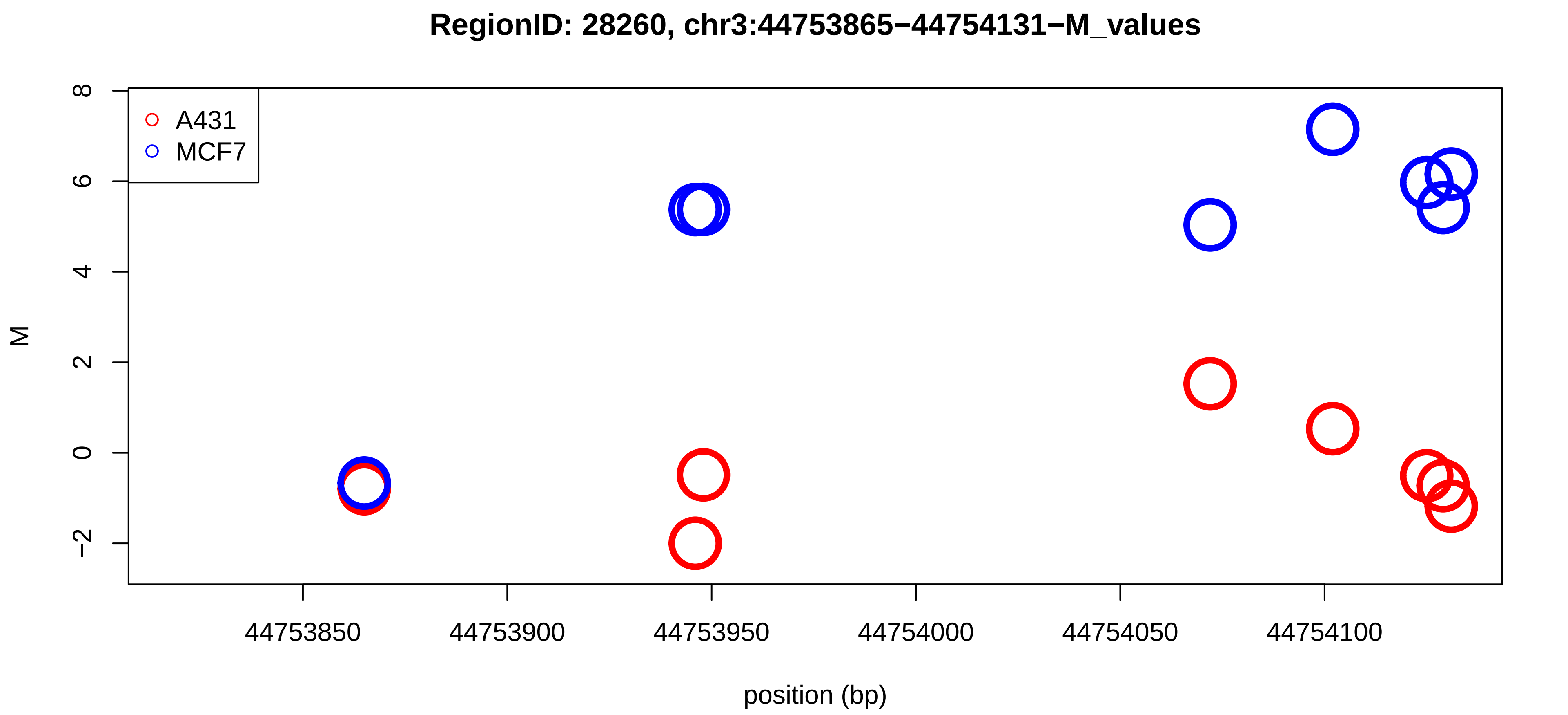
RegionID: 28260, chr3:44753865−44754131−M_values
A431
MCF7
M
8
6
4
2
0
−2
44753850
44753900
44753950
44754000
44754050
44754100
position (bp)

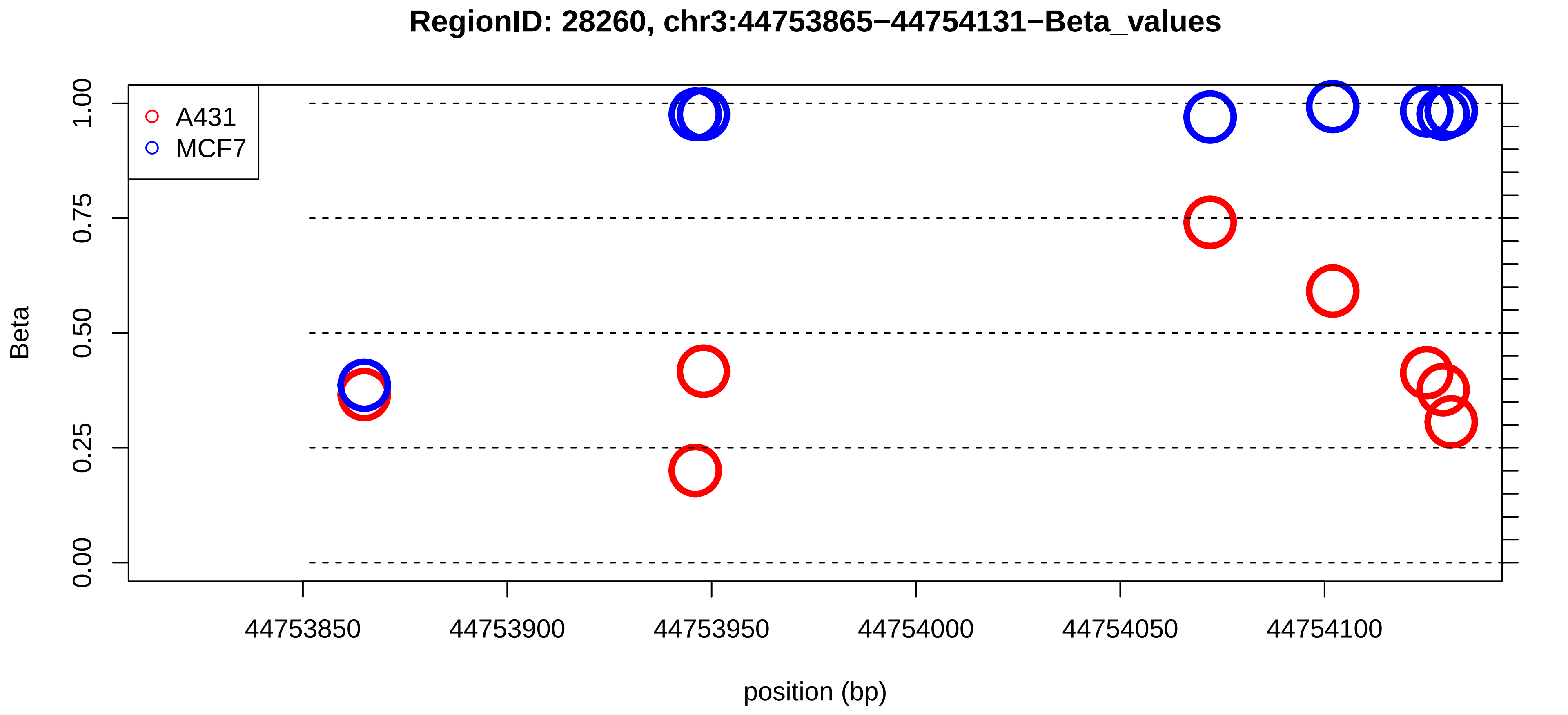
RegionID: 28260, chr3:44753865−44754131−Beta_values
A431
MCF7
Beta
1.00
0.75
0.50
0.25
0.00
44753850
44753900
44753950
44754000
44754050
44754100
position (bp)

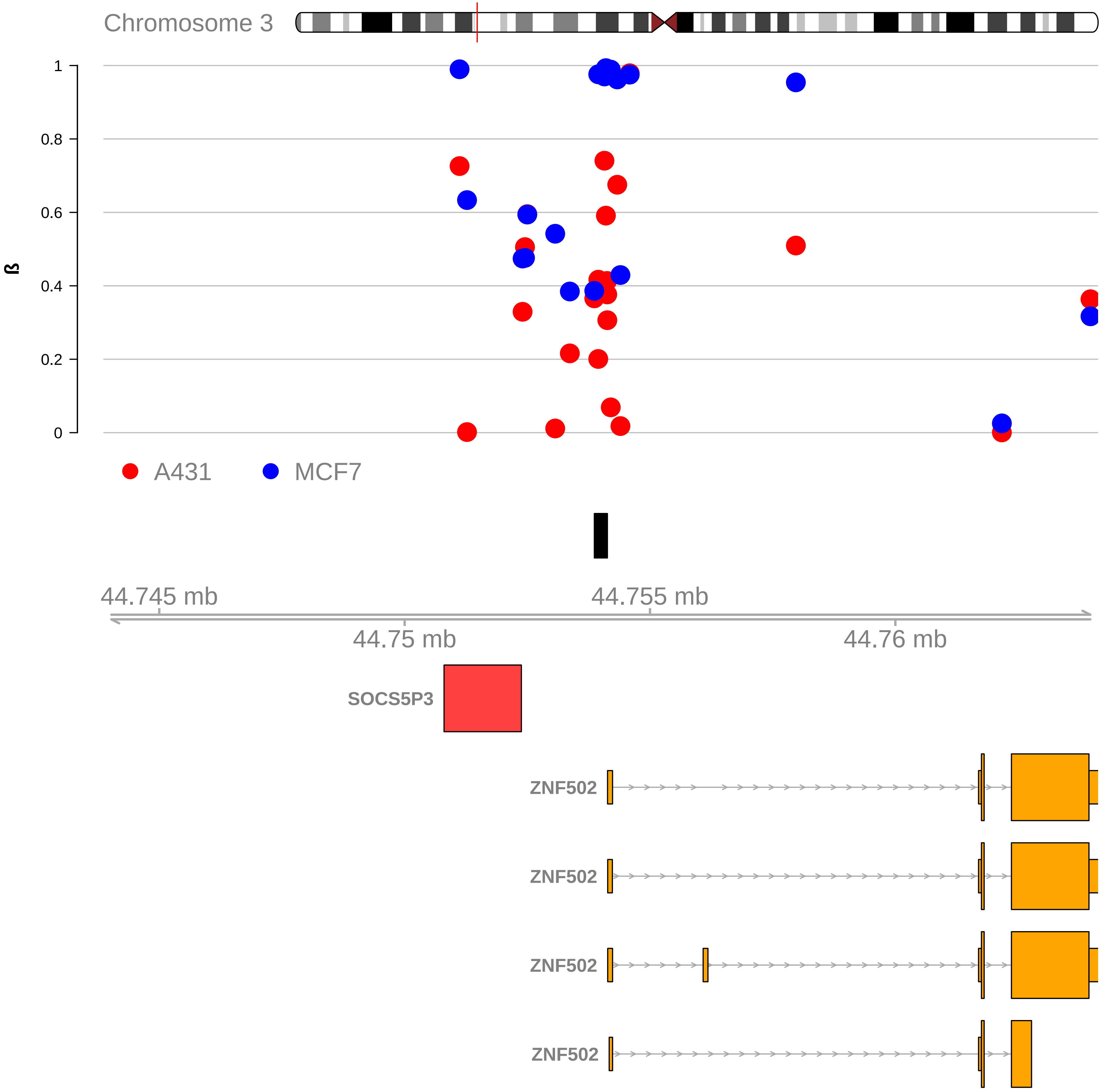

Chromosome 3
1
0.8
0.6
0.4
0.2
0
β
A431
MCF7
44.745 mb
44.755 mb
44.75 mb
44.76 mb
SOCS5P3
ZNF502
ZNF502
ZNF502
ZNF502
